# 1,1′-(Hexane-1,6-di­yl)dipyridinium bis­(hexa­fluoro­phosphate)

**DOI:** 10.1107/S1600536808039639

**Published:** 2008-11-29

**Authors:** Jin-hua Liang, Dong Jin, Xiao-feng Gao, Jin-tang Wang

**Affiliations:** a Department of Applied Chemistry, College of Science, Nanjing University of Technology, Nanjing 210009, People’s Republic of China; b Department of Chemistry and Chemical Engineering, Nanjing University of Technology, Nanjing 210009, People’s Republic of China

## Abstract

The asymmetric unit of the title compound, C_16_H_22_N_2_^2+^·2PF_6_^−^, contains one half-mol­ecule and a hexa­fluoro­phosphate anion. In the crystal structure, inter­molecular C—H⋯F hydrogen bonds link the mol­ecules. The F atoms in the hexa­fluoro­phosphate anion are disordered over two positions and were refined with occupancies of 0.43 (2) and 0.57 (2).

## Related literature

For general background, see: Jared *et al.* (2005 ▶). For bond-length data, see: Allen *et al.* (1987 ▶).
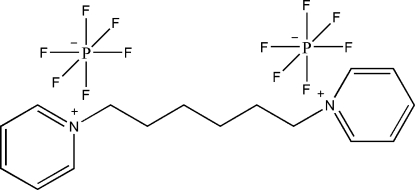


## Experimental

### 

#### Crystal data

C_16_H_22_N_2_^2+^·2PF_6_^−^
*M* *_r_* = 532.30 (3)
Triclinic, 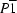

*a* = 7.9140 (16) Å
*b* = 9.2930 (18) Å
*c* = 9.4870 (19) Å
α = 65.13 (3)°
β = 65.46 (3)°
γ = 74.37 (3)°
*V* = 572.0 (3) Å^3^
*Z* = 1
Mo *K*α radiation
μ = 0.29 mm^−1^
*T* = 298 (2) K
0.30 × 0.30 × 0.20 mm

#### Data collection

Enraf–Nonius CAD-4 diffractometer
Absorption correction: ψ scan (North *et al.*, 1968 ▶) *T*_min_ = 0.917, *T*_max_ = 0.944
2172 measured reflections
2014 independent reflections
1499 reflections with *I* > 2σ(*I*)
*R*_int_ = 0.047
3 standard reflections frequency: 120 min intensity decay: none

#### Refinement

*R*[*F* ^2^ > 2σ(*F* ^2^)] = 0.065
*wR*(*F* ^2^) = 0.166
*S* = 1.00
2014 reflections
200 parameters
H-atom parameters constrained
Δρ_max_ = 0.30 e Å^−3^
Δρ_min_ = −0.38 e Å^−3^

### 

Data collection: *CAD-4 Software* (Enraf–Nonius, 1989 ▶); cell refinement: *CAD-4 Software*; data reduction: *XCAD4* (Harms & Wocadlo, 1995 ▶); program(s) used to solve structure: *SHELXS97* (Sheldrick, 2008 ▶); program(s) used to refine structure: *SHELXL97* (Sheldrick, 2008 ▶); molecular graphics: *SHELXTL* (Sheldrick, 2008 ▶); software used to prepare material for publication: *SHELXTL*.

## Supplementary Material

Crystal structure: contains datablocks I, global, x1. DOI: 10.1107/S1600536808039639/hk2581sup1.cif

Structure factors: contains datablocks I. DOI: 10.1107/S1600536808039639/hk2581Isup2.hkl

Additional supplementary materials:  crystallographic information; 3D view; checkCIF report

## Figures and Tables

**Table 1:** Hydrogen-bond geometry (Å, °)

*D*—H⋯*A*	*D*—H	H⋯*A*	*D*⋯*A*	*D*—H⋯*A*
C1—H1*A*⋯F4′^i^	0.93	2.48	3.333 (17)	153
C2—H2*A*⋯F2′^ii^	0.93	2.53	3.267 (18)	137
C3—H3*A*⋯F3′^ii^	0.93	2.47	3.257 (15)	142
C4—H4*A*⋯F1′^iii^	0.93	2.52	3.287 (14)	140

Symmetry codes: (i) 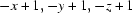; (ii) 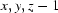; (iii) 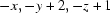.

**Fig. 1.**
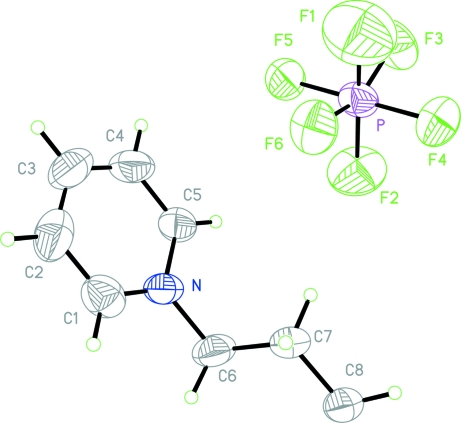
The asymmetric unit of the title compound, with the atom-numbering scheme. Displacement ellipsoids are drawn at the 30% probability level.

**Fig. 2.**
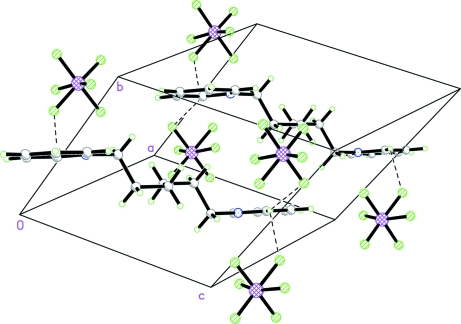
A partial packing diagram of the title compound. Hydrogen bonds are shown as dashed lines.

## supplementary crystallographic information

### Comment

The title compound is a dicationic ionic liquid, which has high thermal stability. Applications of the dicationic ionic liquid are found in biochemistry as well as many areas of chemistry (Jared *et al.*, 2005). We report herein the crystal structure of the title compound.

The asymmetric unit of the title compound (Fig. 1) contains one-half molecule and a hexafluorophosphate molecule, where the bond lengths (Allen *et al.*,  1987) and angles are within normal ranges.

In the crystal structure, intermolecular C-H···F hydrogen bonds (Table 1) link the molecules (Fig. 2), in which they may be effective in the stabilization of the structure.

### Experimental

For the preparation of the title compound, 1,6-dibromide hexane (12.2 g, 0.05 mol) was added to acetonitrile solution (50 ml) of dehydrate pyridine (7.91 g, 0.10 mol) at 353 K. After stirring for 24 h, the mixture was cooled to room temperature and filtered. The solid was washed with ethyl acetate and dried. Then, the solid (2.01 g, 5 mmol) was dissolved in distilled water (50 ml) and potassium hexafluorophosphate (1.84 g, 10 mmol) was added. After stirring at room temperature for 3 h, the colorless solid formed was collected by filtration, washed with distilled water (50 ml) and dried. The product was purified by repeated crystallization. Crystals suitable for X-ray analysis were obtained by slow evaporation of acetone (yield; 3.08 g, 80%, m.p. 513 K).

### Refinement

The F1, F2, F3, F4, F5 and F6 atoms in hexafluorophosphate were disordered over two positions. During the refinement process the disordered atoms were refined with occupancies of 0.43 (2) for F1, F2, F3, F4, F5 , F6 and 0.57 (2) for F1', F2', F3', F4', F5', F6', respectively. H atoms were positioned geometrically, with C-H = 0.93 and 0.97 Å for aromatic and methylene H, respectively, and constrained to ride on their parent atoms with U_iso_(H) = 1.2U_eq_(C).

### Crystal data

**Table:**

C_16_H_22_N_2_^2+^·2P_1_F_6_^–^	*Z* = 1
*M**_r_* = 532.30 (3)	*F*_000_ = 270
Triclinic, *P*1	*D*_x_ = 1.545 Mg m^−^^3^
Hall symbol: -P 1	Melting point: 513 K
*a* = 7.9140 (16) Å	Mo *K*α radiation λ = 0.71073 Å
*b* = 9.2930 (18) Å	Cell parameters from 25 reflections
*c* = 9.4870 (19) Å	θ = 10–12º
α = 65.13 (3)º	µ = 0.29 mm^−^^1^
β = 65.46 (3)º	*T* = 298 (2) K
γ = 74.37 (3)º	Block, colorless
*V* = 572.0 (3) Å^3^	0.30 × 0.30 × 0.20 mm

### Data collection

**Table:**

Enraf–Nonius CAD-4 diffractometer	*R*_int_ = 0.047
Radiation source: fine-focus sealed tube	θ_max_ = 25.1º
Monochromator: graphite	θ_min_ = 2.4º
*T* = 298(2) K	*h* = −8→9
ω/2θ scans	*k* = −9→10
Absorption correction: ψ scan(North *et al.*, 1968)	*l* = 0→11
*T*_min_ = 0.917, *T*_max_ = 0.944	3 standard reflections
2172 measured reflections	every 120 min
2014 independent reflections	intensity decay: none
1499 reflections with *I* > 2σ(*I*)	

### Refinement

**Table:**

Refinement on *F*^2^	Secondary atom site location: difference Fourier map
Least-squares matrix: full	Hydrogen site location: inferred from neighbouring sites
*R*[*F*^2^ > 2σ(*F*^2^)] = 0.065	H-atom parameters constrained
*wR*(*F*^2^) = 0.166	*w* = 1/[σ^2^(*F*_o_^2^) + (0.06*P*)^2^ + 0.95*P*] where *P* = (*F*_o_^2^ + 2*F*_c_^2^)/3
*S* = 1.01	(Δ/σ)_max_ < 0.001
2014 reflections	Δρ_max_ = 0.30 e Å^−^^3^
200 parameters	Δρ_min_ = −0.38 e Å^−^^3^
Primary atom site location: structure-invariant direct methods	Extinction correction: none

### Special details

**Table:**

Geometry. All e.s.d.'s (except the e.s.d. in the dihedral angle between two l.s. planes) are estimated using the full covariance matrix. The cell e.s.d.'s are taken into account individually in the estimation of e.s.d.'s in distances, angles and torsion angles; correlations between e.s.d.'s in cell parameters are only used when they are defined by crystal symmetry. An approximate (isotropic) treatment of cell e.s.d.'s is used for estimating e.s.d.'s involving l.s. planes.
Refinement. Refinement of *F*^2^ against ALL reflections. The weighted *R*-factor *wR* and goodness of fit *S* are based on *F*^2^, conventional *R*-factors *R* are based on *F*, with *F* set to zero for negative *F*^2^. The threshold expression of *F*^2^ > σ(*F*^2^) is used only for calculating *R*-factors(gt) *etc*. and is not relevant to the choice of reflections for refinement. *R*-factors based on *F*^2^ are statistically about twice as large as those based on *F*, and *R*- factors based on ALL data will be even larger.

### Fractional atomic coordinates and isotropic or equivalent isotropic displacement parameters (Å^2^)

**Table:**

	*x*	*y*	*z*	*U*_iso_*/*U*_eq_	Occ. (<1)
P	0.16995 (14)	0.79005 (13)	0.78487 (13)	0.0539 (4)	
N	0.6355 (4)	0.7536 (4)	0.2462 (4)	0.0472 (8)	
F1	−0.006 (3)	0.696 (3)	0.842 (2)	0.156 (6)	0.43 (2)
F2	0.369 (2)	0.8457 (19)	0.721 (2)	0.104 (5)	0.43 (2)
F3	0.034 (2)	0.8894 (16)	0.8982 (15)	0.091 (4)	0.43 (2)
F4	0.215 (2)	0.645 (2)	0.939 (2)	0.076 (4)	0.43 (2)
F5	0.126 (2)	0.936 (2)	0.635 (2)	0.078 (4)	0.43 (2)
F6	0.223 (3)	0.677 (3)	0.684 (2)	0.076 (4)	0.43 (2)
F1'	−0.0338 (9)	0.7809 (16)	0.8155 (14)	0.118 (4)	0.57 (2)
F2'	0.375 (2)	0.785 (2)	0.775 (2)	0.144 (5)	0.57 (2)
F3'	0.127 (2)	0.9036 (12)	0.8910 (13)	0.098 (3)	0.57 (2)
F4'	0.150 (2)	0.6338 (18)	0.9501 (17)	0.090 (4)	0.57 (2)
F5'	0.195 (2)	0.9495 (17)	0.6211 (17)	0.094 (4)	0.57 (2)
F6'	0.2820 (19)	0.6854 (19)	0.6687 (18)	0.082 (4)	0.57 (2)
C1	0.6255 (6)	0.6893 (5)	0.1468 (5)	0.0607 (11)	
H1A	0.7227	0.6159	0.1113	0.073*	
C2	0.4732 (7)	0.7312 (6)	0.0972 (6)	0.0735 (13)	
H2A	0.4661	0.6866	0.0288	0.088*	
C3	0.3292 (6)	0.8420 (6)	0.1517 (6)	0.0751 (14)	
H3A	0.2260	0.8736	0.1179	0.090*	
C4	0.3402 (6)	0.9030 (6)	0.2537 (6)	0.0721 (13)	
H4A	0.2428	0.9751	0.2913	0.087*	
C5	0.4934 (5)	0.8603 (5)	0.3029 (5)	0.0543 (10)	
H5A	0.5003	0.9029	0.3730	0.065*	
C6	0.7977 (5)	0.7070 (5)	0.3014 (5)	0.0580 (10)	
H6A	0.8387	0.8022	0.2896	0.070*	
H6B	0.8998	0.6558	0.2307	0.070*	
C7	0.7536 (5)	0.5942 (5)	0.4801 (5)	0.0563 (10)	
H7A	0.7184	0.4969	0.4908	0.068*	
H7B	0.6475	0.6434	0.5502	0.068*	
C8	0.9189 (5)	0.5519 (5)	0.5406 (5)	0.0602 (11)	
H8A	0.9628	0.6498	0.5183	0.072*	
H8B	0.8761	0.4963	0.6595	0.072*	

### Atomic displacement parameters (Å^2^)

**Table:**

	*U*^11^	*U*^22^	*U*^33^	*U*^12^	*U*^13^	*U*^23^
P	0.0470 (6)	0.0550 (6)	0.0538 (6)	0.0051 (4)	−0.0114 (5)	−0.0263 (5)
N	0.0332 (15)	0.0466 (17)	0.0475 (17)	−0.0002 (13)	−0.0093 (13)	−0.0107 (14)
F1	0.136 (9)	0.160 (11)	0.165 (9)	−0.050 (8)	−0.033 (7)	−0.049 (8)
F2	0.082 (6)	0.104 (7)	0.133 (9)	−0.045 (5)	−0.035 (6)	−0.030 (6)
F3	0.101 (8)	0.077 (5)	0.074 (5)	0.016 (5)	−0.007 (5)	−0.048 (4)
F4	0.085 (8)	0.067 (5)	0.077 (7)	−0.003 (6)	−0.043 (6)	−0.016 (4)
F5	0.070 (6)	0.080 (7)	0.059 (5)	0.015 (5)	−0.024 (5)	−0.015 (4)
F6	0.097 (9)	0.078 (5)	0.083 (8)	−0.014 (6)	−0.038 (7)	−0.046 (5)
F1'	0.041 (3)	0.113 (7)	0.173 (7)	−0.002 (3)	−0.039 (3)	−0.027 (5)
F2'	0.118 (6)	0.180 (10)	0.147 (8)	−0.033 (7)	−0.069 (6)	−0.036 (7)
F3'	0.126 (7)	0.088 (4)	0.086 (4)	0.000 (5)	−0.021 (5)	−0.058 (3)
F4'	0.090 (7)	0.068 (4)	0.074 (4)	0.010 (5)	−0.009 (5)	−0.022 (3)
F5'	0.124 (8)	0.077 (4)	0.069 (4)	−0.014 (6)	−0.023 (6)	−0.023 (3)
F6'	0.074 (6)	0.078 (5)	0.075 (4)	0.002 (4)	0.003 (4)	−0.043 (3)
C1	0.064 (3)	0.052 (2)	0.057 (2)	0.0000 (19)	−0.015 (2)	−0.022 (2)
C2	0.084 (3)	0.086 (3)	0.066 (3)	−0.017 (3)	−0.040 (3)	−0.023 (3)
C3	0.051 (3)	0.095 (4)	0.066 (3)	−0.015 (2)	−0.031 (2)	−0.002 (3)
C4	0.042 (2)	0.083 (3)	0.066 (3)	0.014 (2)	−0.014 (2)	−0.021 (2)
C5	0.050 (2)	0.056 (2)	0.053 (2)	0.0118 (18)	−0.0171 (18)	−0.0268 (19)
C6	0.0340 (19)	0.066 (3)	0.066 (3)	−0.0019 (17)	−0.0187 (18)	−0.017 (2)
C7	0.0339 (19)	0.074 (3)	0.057 (2)	0.0055 (18)	−0.0156 (17)	−0.027 (2)
C8	0.044 (2)	0.082 (3)	0.060 (2)	0.009 (2)	−0.0230 (19)	−0.035 (2)

### Geometric parameters (Å, °)

**Table:**

P—F1'	1.535 (7)	C3—C4	1.350 (7)
P—F6	1.567 (19)	C3—H3A	0.9300
P—F2'	1.575 (15)	C4—C5	1.379 (6)
P—F3	1.582 (11)	C4—H4A	0.9300
P—F2	1.583 (14)	C5—N	1.372 (5)
P—F5	1.588 (16)	C5—H5A	0.9300
P—F6'	1.604 (14)	N—C6	1.476 (5)
P—F4'	1.611 (14)	C6—C7	1.518 (6)
P—F4	1.612 (18)	C6—H6A	0.9700
P—F5'	1.617 (14)	C6—H6B	0.9700
P—F1	1.619 (15)	C7—C8	1.534 (5)
P—F3'	1.631 (9)	C7—H7A	0.9700
C1—N	1.347 (5)	C7—H7B	0.9700
C1—C2	1.375 (6)	C8—C8^i^	1.518 (7)
C1—H1A	0.9300	C8—H8A	0.9700
C2—C3	1.397 (7)	C8—H8B	0.9700
C2—H2A	0.9300		
			
F1'—P—F6	85.7 (10)	F4—P—F1	83.6 (8)
F1'—P—F2'	173.4 (6)	F5'—P—F1	116.5 (8)
F6—P—F2'	95.7 (10)	F1'—P—F3'	97.5 (6)
F1'—P—F3	70.4 (7)	F6—P—F3'	176.5 (11)
F6—P—F3	156.1 (13)	F2'—P—F3'	80.9 (7)
F2'—P—F3	107.8 (9)	F2—P—F3'	78.8 (9)
F1'—P—F2	164.7 (6)	F5—P—F3'	92.5 (8)
F6—P—F2	98.4 (9)	F6'—P—F3'	160.7 (10)
F3—P—F2	105.1 (10)	F4'—P—F3'	90.4 (7)
F1'—P—F5	76.2 (6)	F4—P—F3'	86.1 (8)
F6—P—F5	89.5 (10)	F5'—P—F3'	88.0 (6)
F2'—P—F5	110.2 (6)	F1—P—F3'	112.6 (7)
F3—P—F5	86.5 (8)	N—C1—C2	120.6 (4)
F2—P—F5	89.1 (7)	N—C1—H1A	119.7
F1'—P—F6'	101.7 (9)	C2—C1—H1A	119.7
F2'—P—F6'	80.1 (9)	C1—C2—C3	118.7 (4)
F3—P—F6'	172.1 (12)	C1—C2—H2A	120.7
F2—P—F6'	82.4 (8)	C3—C2—H2A	120.7
F5—P—F6'	91.2 (9)	C4—C3—C2	119.8 (4)
F1'—P—F4'	86.0 (5)	C4—C3—H3A	120.1
F6—P—F4'	88.5 (9)	C2—C3—H3A	120.1
F2'—P—F4'	87.5 (6)	C3—C4—C5	121.1 (4)
F3—P—F4'	88.2 (7)	C3—C4—H4A	119.4
F2—P—F4'	108.7 (6)	C5—C4—H4A	119.4
F5—P—F4'	162.2 (6)	N—C5—C4	118.6 (4)
F6'—P—F4'	91.8 (7)	N—C5—H5A	120.7
F1'—P—F4	104.3 (6)	C4—C5—H5A	120.7
F6—P—F4	91.8 (10)	C1—N—C5	121.2 (3)
F2'—P—F4	69.2 (7)	C1—N—C6	120.7 (3)
F3—P—F4	92.5 (9)	C5—N—C6	118.1 (3)
F2—P—F4	90.3 (7)	N—C6—C7	112.5 (3)
F5—P—F4	178.6 (11)	N—C6—H6A	109.1
F6'—P—F4	90.0 (9)	C7—C6—H6A	109.1
F1'—P—F5'	95.7 (6)	N—C6—H6B	109.1
F6—P—F5'	93.0 (9)	C7—C6—H6B	109.1
F2'—P—F5'	90.7 (6)	H6A—C6—H6B	107.8
F3—P—F5'	91.0 (7)	C6—C7—C8	112.7 (3)
F2—P—F5'	69.5 (6)	C6—C7—H7A	109.0
F6'—P—F5'	89.2 (8)	C8—C7—H7A	109.0
F4'—P—F5'	177.8 (7)	C6—C7—H7B	109.0
F4—P—F5'	159.8 (6)	C8—C7—H7B	109.0
F6—P—F1	69.9 (12)	H7A—C7—H7B	107.8
F2'—P—F1	149.1 (8)	C8^i^—C8—C7	113.4 (4)
F3—P—F1	87.2 (7)	C8^i^—C8—H8A	108.9
F2—P—F1	166.6 (10)	C7—C8—H8A	108.9
F5—P—F1	97.2 (9)	C8^i^—C8—H8B	108.9
F6'—P—F1	85.7 (11)	C7—C8—H8B	108.9
F4'—P—F1	65.6 (7)	H8A—C8—H8B	107.7

Symmetry codes: (i) −*x*+2, −*y*+1, −*z*+1.

### Hydrogen-bond geometry (Å, °)

**Table:**

*D*—H···*A*	*D*—H	H···*A*	*D*···*A*	*D*—H···*A*
C1—H1A···F4'^ii^	0.93	2.48	3.333 (17)	153
C2—H2A···F2'^iii^	0.93	2.53	3.267 (18)	137
C3—H3A···F3'^iii^	0.93	2.47	3.257 (15)	142
C4—H4A···F1'^iv^	0.93	2.52	3.287 (14)	140

Symmetry codes: (ii) −*x*+1, −*y*+1, −*z*+1; (iii) *x*, *y*, *z*−1; (iv) −*x*, −*y*+2, −*z*+1.
